# Does a decannulation protocol exist in COVID-19 patients? The importance of working in a multiprofessional team

**DOI:** 10.1007/s44250-023-00031-z

**Published:** 2023-04-13

**Authors:** E. Cavalli, G. Belfiori, G. Molinari, A. Peghetti, A. Zanoni, E. Chinelli

**Affiliations:** 1 Physical Medicine and Rehabilitation Unit, IRCCS Azienda Ospedaliero-Universitaria di Bologna, Via Albertoni N°15, 40138 Bologna, Italy; 2 Otolaryngology and Audiology Unit, IRCCS Azienda Ospedaliero-Universitaria di Bologna, Via Albertoni N°15, 40138 Bologna, Italy; 3 IRCCS Azienda Ospedaliero-Universitaria di Bologna, Via Albertoni N°15, 40138 Bologna, Italy; 4 Anesthesia and Intensive Care in Local, Regional and National Emergencies and in Major Abdominal Surgery, IRCCS Azienda Ospedaliero-Universitaria di Bologna, Via Albertoni N°15, 40138 Bologna, Italy

## Abstract

As a Covid Hub in Emilia Romagna, we have experienced an increasing number of tracheostomized patients, prompting us to develop a standardized decannulation protocol for COVID-19 ARDS patients. Currently, there are no guidelines or protocols for decannulation in this population, and few studies have investigated the early outcomes of tracheostomy in COVID-19 patients, with no detailed analysis of the decannulation process. We recognized the importance of mutual reliance among our team members and the significant achievements we made compared to previous decannulation methods. Through the optimization of the decannulation process, we identified a clear, safe, and repeatable method based on clinical best practice and literature evidence. We decided to implement an existing standardized decannulation protocol, which was originally designed for severe brain-damaged patients, due to the growing number of COVID-19 patients with tracheostomy. This protocol was designed for daily practice and aimed to provide a uniform approach to using devices like fenestrated cannulas, speaking valves, and capping. The results of our implementation include:

- expanding the applicability of the protocol beyond severe brain-damaged patients to different populations and settings (in this case, patients subjected to a long period of sedation and invasive ventilation)
- early activation of speech therapy to facilitate weaning from the cannula and recovery of physiological swallowing and phonation
- early activation of otolaryngologist evaluation to identify organic problems related to prolonged intubation, tracheostomy, and ventilation and address proper speech therapy treatment
- activation of more fluid and effective management paths for decannulation with a multiprofessional team.

## Introduction

COVID-19 infection can result in severe clinical symptoms in approximately 5% of cases, including respiratory failure, septic shock, and multiple organ dysfunction or failure [1]. Tracheostomy for COVID-19 pneumonia was typically delayed, with a timing of more than 14 days after intubation, similar to what has been described in the literature [2–6]. Early tracheostomy had unfavorable outcomes during the initial stages of the COVID-19 pandemic, with high mortality rates, so tracheostomy was delayed until patients showed signs of recovery and had a higher chance of success. At the time of the study, our patient population exhibited severe ARDS related to COVID-19, with an inability to hold low PEEP or a supine position, or periods of apnea during tracheostomy, and there was a risk of virus transmission to healthcare workers during the procedure, as well as a significant risk of barotrauma [2, 5, 7–9].

Tracheostomy in the ICU plays a role in weaning patients who are predicted to require prolonged ventilation as it facilitates weaning off from sedation and airway management, and reduces the risk of complications affecting the tracheal and chordal planes from prolonged intubation [10]. Current evidence shows no difference in outcomes between surgical and percutaneous tracheostomy [7, 11–15]. Of ICU patients who are intubated and then tracheostomized, 62% present with dysphagia [16–18]. The presence of the cuffed cannula alters the physiological mechanisms of swallowing and phonation due to the loss of sensitivity, incoordination of laryngeal closure, esophageal obstruction, and potential for damage to the trachea [19–23]. Neurological deficits and peripheral nerves damages can also have a significant impact on swallowing [24]. The ultimate goal of decannulation is to restore mechanisms of swallowing, vocal cord function, and speech [19, 25]. The risk of complications related to tracheostomy is proportional to the amount of time that the patient has the cannula [26].

During the acute COVID-19 emergency phase, the closure of the Intensive Rehabilitation Department in our Center (IRCCS Azienda Ospedaliero-Universitaria di Bologna) and the lack of experience in handling tracheostomized patients in other departments had exposed patients to risks, necessitating the adoption of a standardized decannulation protocol.

## Materials and methods

There are only five studies [2, 3, 7, 27–29] that have been examined in the early outcomes of COVID-19 patients with tracheostomy, and none of them include a detailed analysis of the decannulation process.

**Fig. 1:**
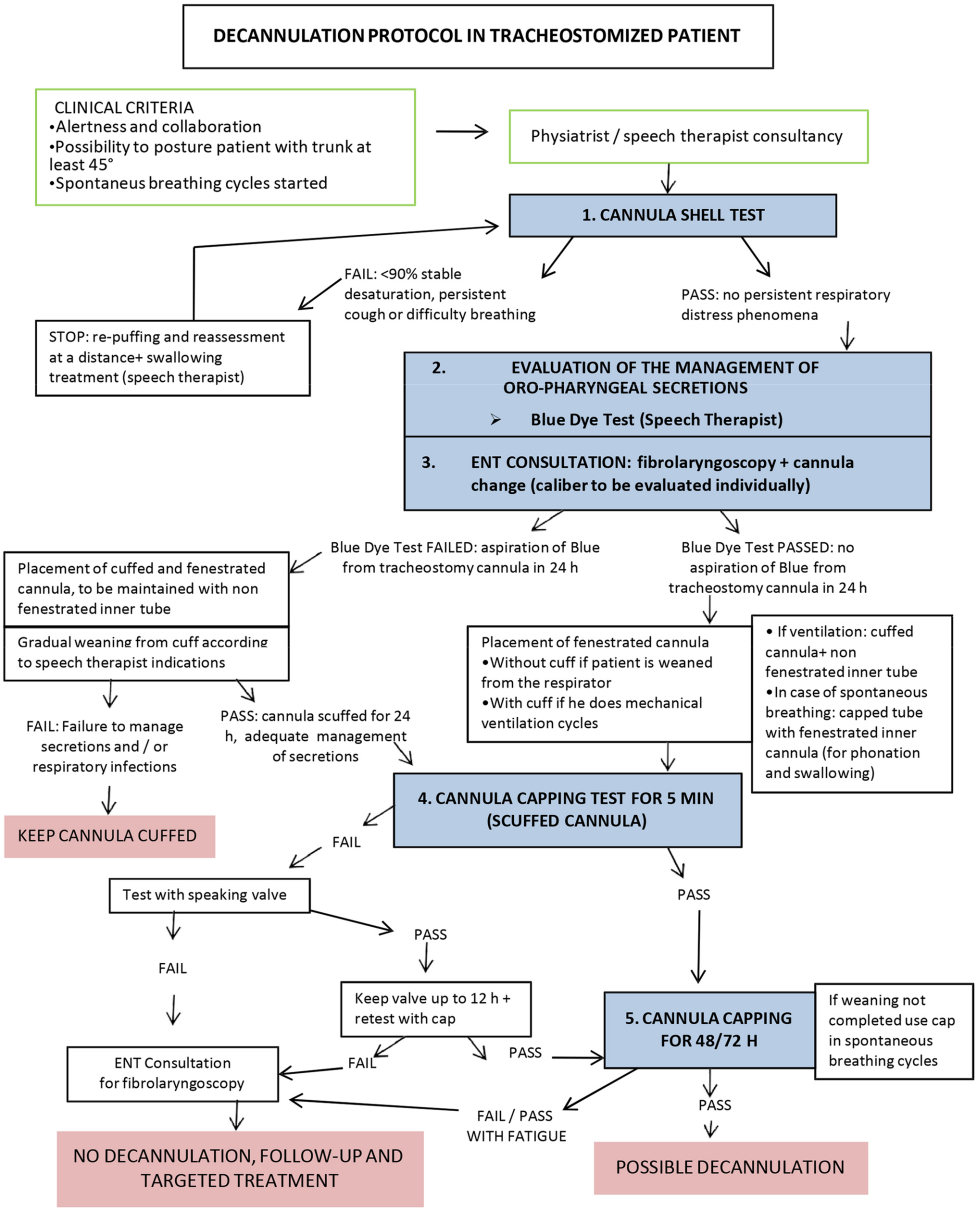
Speech therapy protocol in use at Covid Intensive Care— MD Zanoni A., SLT Belfiori G.—, MD Chinelli E., MD Cavalli E., MD Molinari G. see Bargellesi S, Reverberi C., (April 2013), Giornale Italiano di Medicina Riabilitativa, Vol. 27, N. 1

For this reason, we adapted an existing protocol that was developed for patients with severe acquired brain injury [30], and created a new protocol that aims to optimize the decannulation process and identify any complications early on (such as laryngeal lesions, chordal paralysis, granulomas, necrosis, etc.). Our goal was to achieve the best practices in terms of safety and prevention of complications from tracheostomy. Additionally, we aimed to have a unified approach in the use of devices like fenestrated cannulas, speaking valves, and capping [5, 11, 20, 21, 31, 32]. Based on these considerations, we developed a new cannula weaning protocol for patients who had recovered from severe COVID-19 infection (as illustrated in Fig. 1). We contacted the first two authors of the existing protocol and informed them of our intent to implement the algorithm in order to improve the clinical quality of care and safety in managing the weaning process from the tracheostomy cannula in patients with post-COVID-19 ARDS. The implementation period extended from March 2021 to November 2021, during which 148 patients were admitted to the ICU.

We included patients who were adults aged 18 years or older, diagnosed with acute respiratory failure due to COVID-19 pneumonia, and in need of orotracheal intubation and tracheostomy for weaning.

We excluded patients who were transferred to another hospital and had lost outcome data, as well as those who had developed clinical complications that were unsuitable for continuing the decannulation process and following the protocol.

## Results

We selected a sample of 22 patients who met the inclusion criteria, 8 of whom were excluded from the study due to non-compliance with the protocol, including 2 patients who were transferred to another hospital. The success of the protocol was evaluated based on various parameters, including the number of patients who successfully completed each step (14 patients), the time to decannulation (12 days), the average intubation time (24 days—the number of days on ventilation before tracheo-stomy), and the average time between tracheostomy and the first cannula change (20 days). At discharge, all patients passed the cannula scuffing test and were able to feed orally. Of the total number of patients who underwent the protocol, 85.7% had a single cannula change and the remaining 14.3% required two cannula changes. Additionally, 71.4% passed the Blue Dye Test and 28.6% failed. During the otolaryngologist evaluation, complications were found in 1 patient with suprastomal granuloma, 6 patients with arytenoid edema, 1 patient with hypomobility of one vocal cord, and 2 patients with bellows deficit dysphonia.

Our findings include:

1. The applicability of the protocol originally designed for severe brain-damaged patients can be extended to different populations and settings, such as patients who had undergone long periods of sedation and invasive ventilation.
2. Early activation of speech therapy according to the protocol facilitated decannulation and aided in the recovery of physiological swallowing and phonation.
3. Early activation of otolaryngologist evaluation according to the protocol facilitated identification of organic problems related to prolonged intubation, tracheostomy and ventilation, and allowed for proper speech therapy treatment.
4. More efficient management paths for decannulation with a multiprofessional team were activated, resulting in a smoother and more effective process.

## Discussion

Although there is a considerable amount of literature on sedation and weaning management, decannulation criteria in the ICU are not standardized, and logopedic and motor rehabilitation specialists do not often collaborate with anesthesiologists in patient care. Similarly, otolaryngologists are only involved in patient care if problems are found during decannulation. As previously noted, weaning COVID-19 pneumonia patients is frequently managed with tracheostomy due to prolonged bed rest and critical illness, which is related to pulmonary and neurological outcomes. Our tracheostomy decannulation protocol is triggered by the improvement of a patient's clinical condition, resolution of the acute phase, adequate gas exchange, and the ability to sustain spontaneous breathing despite the need for short cycles of ventilatory support (FiO2 < 40% and low PEEP) to complete weaning. During this early phase, a patient undergoes both speech therapy and otolaryngology evaluation and, if possible, a smaller cannula eventually fenestrated is positioned (as shown in Fig. 1).

We believe that the strength of this protocol lies in the early activation of speech therapy, which reduces the time that the patient requires a cuffed cannula and minimizes the impact on physiological swallowing and phonation. The early evaluation by otolaryngologists also helps exclude any organic problems related to prolonged intubation and tracheostomy and allows for appropriate speech therapy treatment [33, 34]. We believe that a protocol originally designed for severe brain-damaged patients and adapted to COVID-19 patients could be applied to different populations in order to achieve the best practice in the decannulation process.

Implications for future practice and research include standardizing the decannulation approach according to evidence-based models and guidelines, improving nursing and medical management skills, including early screening of patients with dysphagia and cognitive problems related to prolonged sedation, and emphasizing the crucial role of speech therapists in patient management during the pandemic. Further research is needed to explore the role of speech therapists in the decannulation process in other intensive therapies [35]. Larger studies at different centers are necessary to confirm the results and establish the protocol's applicability in COVID-19 patients and different clinical settings. The protocol's adaptability to different populations and settings, including patients with COVID-19 pneumonia, highlights its potential for widespread use in a variety of clinical settings.

## Limitations

Small samples due to exclusion from the protocol of some patients moved to a Rehabilitation Hospital outside our Centre (IRCCS Azienda Ospedaliero-Universitaria di Bologna). The closure of the Intensive Rehabilitation Department during the acute COVID-19 emergency phase and the lack of experience in handling tracheostomized patients in other departments were in fact a good reason to improve a decannulation process in ICU.

## Data Availability

Data that support the findings of this study are not openly available due to reasons of sensitivity e.g. human data and are available from the corresponding author upon sensitive request and however, data will be transmitted in partial and filtered ways so that the patient's privacy is not violated.

## Abbreviations

ICU: Intensive Care Unit
ENT: Ear, Nose, Throat
